# Supplementary material for: Usability of a novel lateral flow assay for the point-of-care detection of Neisseria gonorrhoeae: A qualitative time-series assessment among healthcare workers in South Africa
Source: PLoS One. 2023 Jun 2;18(6):e0286666. doi: 10.1371/journal.pone.0286666 (PMC10237465; doi:10.1371/journal.pone.0286666)
Supplement: S1 File — (PDF) [file pone.0286666.s001.pdf]

File S1. FIND NG-LFA Testing and Reader- Set Up Instructions (QuickCards).

Reprinted under a CC BY license, with permission from DCN, original copyright 2022.

FIND POC NG Test Procedure Card for Testing Vaginal Swab Samples

1. Enter sample info on log sheet.
2. Fill medicine dropper to **<u>0.8 mL</u>** (line above 0.8) with Extraction Buffer.

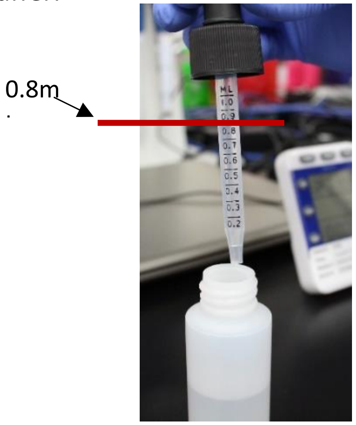

3. Add the 0.8 mL of Extraction Buffer to clean sample tube.

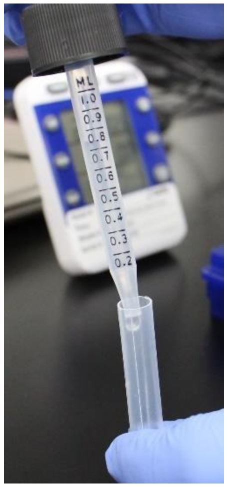

4. Insert the patient swab into the sample tube with extraction buffer.

   Pinch the bottom of the tube and **<u>twirl the swab</u> <u>10 times</u>** to extract the cells "like a sponge."

   Move the swab up and down to mix well.

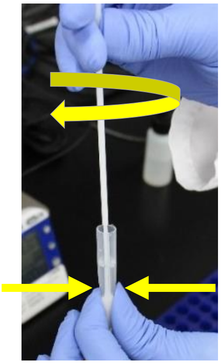

5. Wait **<u>1 minute</u>** with swab remaining in the tube.

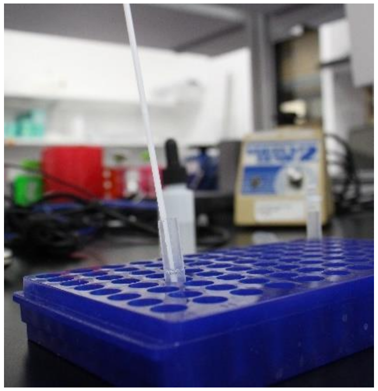

6. Remove the test cassette from the foil pouch.

   Write the sample ID on the cassette below the sample port.

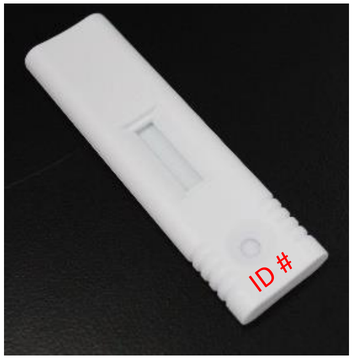

7. **Again** pinch and twirl the swab 10 times, moving the swab up and down to mix.

   <u>Pinch the tube at the top</u> to squeeze the swab as you withdraw it.

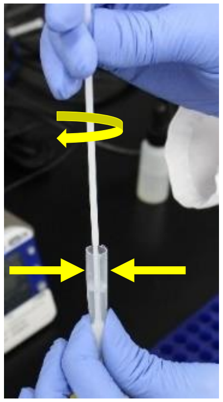

8. Insert a dropper cap onto the sample tube. **<u>Twist</u>** the cap onto the tube to seal it tight.

   Do not touch tip!

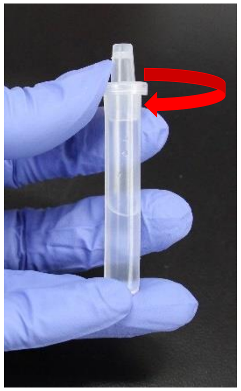

9. Add **<u>4 DROPS</u>** of the sample to the **circular sample port** of cassette.

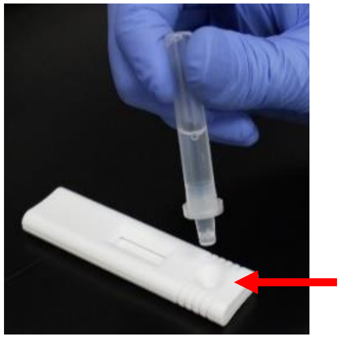

Allow test cassette to sit for **<u>20 minutes</u>** (timer) then use the FIND POC Reader.

Enter test result on log sheet.

DCN DX

FIND POC NG Test Procedure Card for Testing Urine Samples

1. Enter sample info on log sheet.
2. Fill medicine dropper to **<u>0.8 mL</u>** (line above 0.8) with Extraction Buffer.

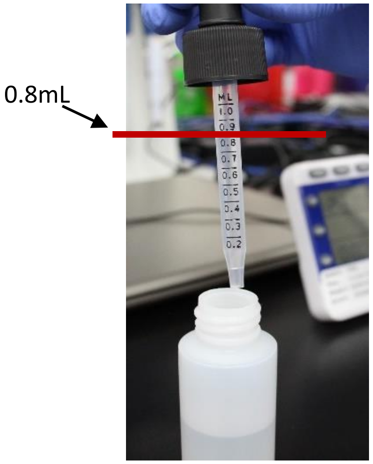

3. Add the 0.8 mL of Extraction Buffer to clean sample tube.

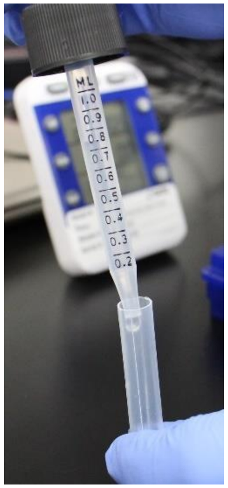

4. Fill disposable pipette to **<u>0.5 mL</u>** (halfway) with urine specimen.

   Add the 0.5 mL of urine to the sample tube.

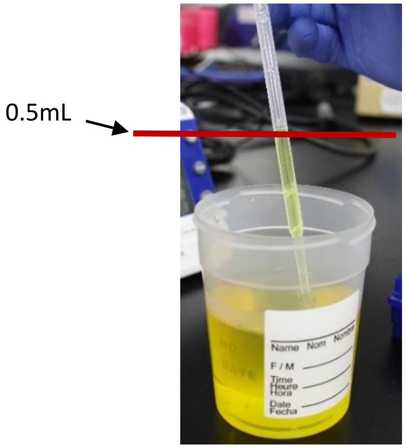

5. Mix sample up & down with transfer pipette **<u>3 times</u>**.

   Wait **<u>1 minute</u>** with urine in the mixed buffer tube.

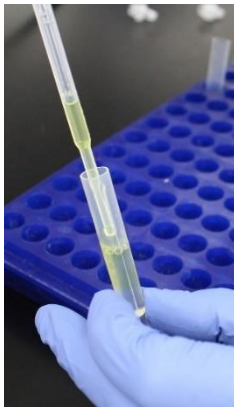

6. Remove the test cassette from the foil pouch.

   Write the sample ID on the cassette below the sample port.

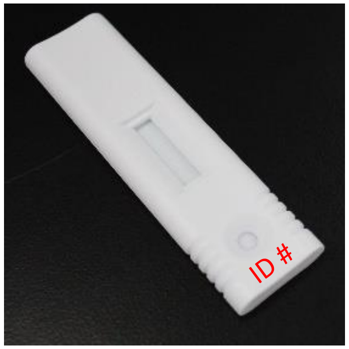

7. Insert a dropper cap onto the sample tube. **<u>Twist</u>** the cap onto the tube to seal it tight.

   Do not touch tip!

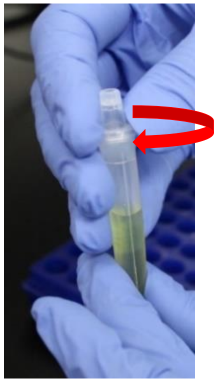

8. Add **<u>4 DROPS</u>** of the sample from the tube to the **circular sample port** of cassette.

   USE DROPPER CAP NOT PIPET!

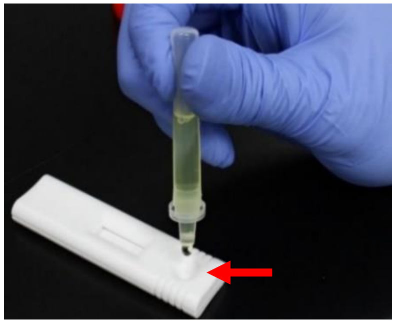

9. Allow test cassette test to sit for **<u>20 minutes</u>** (timer) then use the FIND POC Reader.

   Enter test result on log sheet.

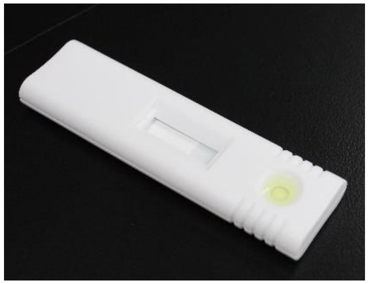

FIND POC NG: FIND POC Reader Start-Up, Reading POCT Cassettes

Reader set up:

10. **TURN ON** the battery pack (press button to see blue lights).

    Make sure battery is connect to POC Reader with grey USB cord.

11. **TURN ON** the FIND POC Reader by pressing the power button and hold for 2 seconds, green light will turn ON.

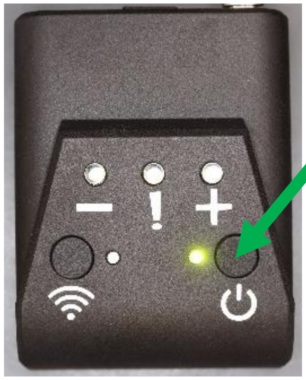

Power Button

NOTE: If battery is drained, plug POC Reader into laptop USB port.

12. Insert cassette into the POC Reader. All three lights will flash to show a cassette is detected. Scan should take < 5 seconds.

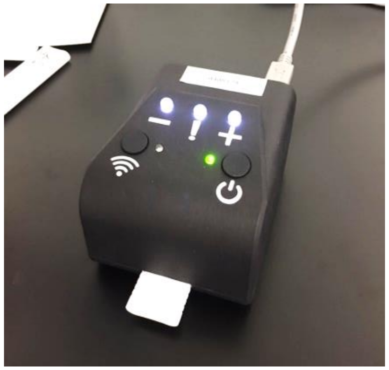

4. The test result will be shown by a single light:

NEGATIVE (-)	ERROR (!)	POSITIVE (+)
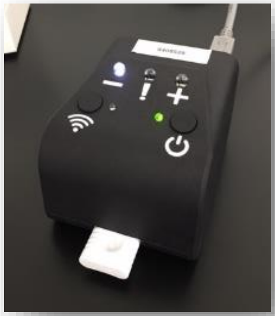	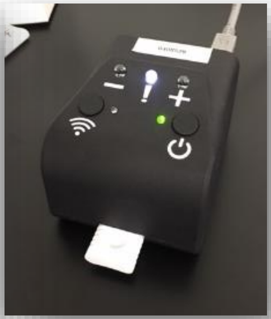	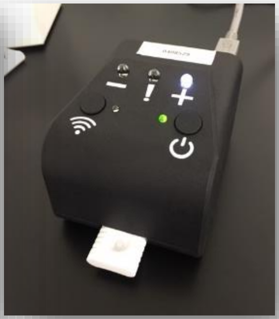

5. Record results in Testing Log sheet.

6. Remove cassette from the POC Reader.

7. **TURN OFF** POC Reader (press power button for 2 seconds). Green light will turn OFF.

IF ERROR (!) occurs, try removing cassette, check orientation, and scan again.

If ERROR persists after re-scan, note in Testing Log and move onto next sample.

If ERROR persists with next sample, contact Study Manager.
