# Supplementary figures and images for: Usability of a novel lateral flow assay for the point-of-care detection of Neisseria gonorrhoeae: A qualitative time-series assessment among healthcare workers in South Africa
Source: PLoS One. 2023 Jun 2;18(6):e0286666. doi: 10.1371/journal.pone.0286666 (PMC10237465; doi:10.1371/journal.pone.0286666)

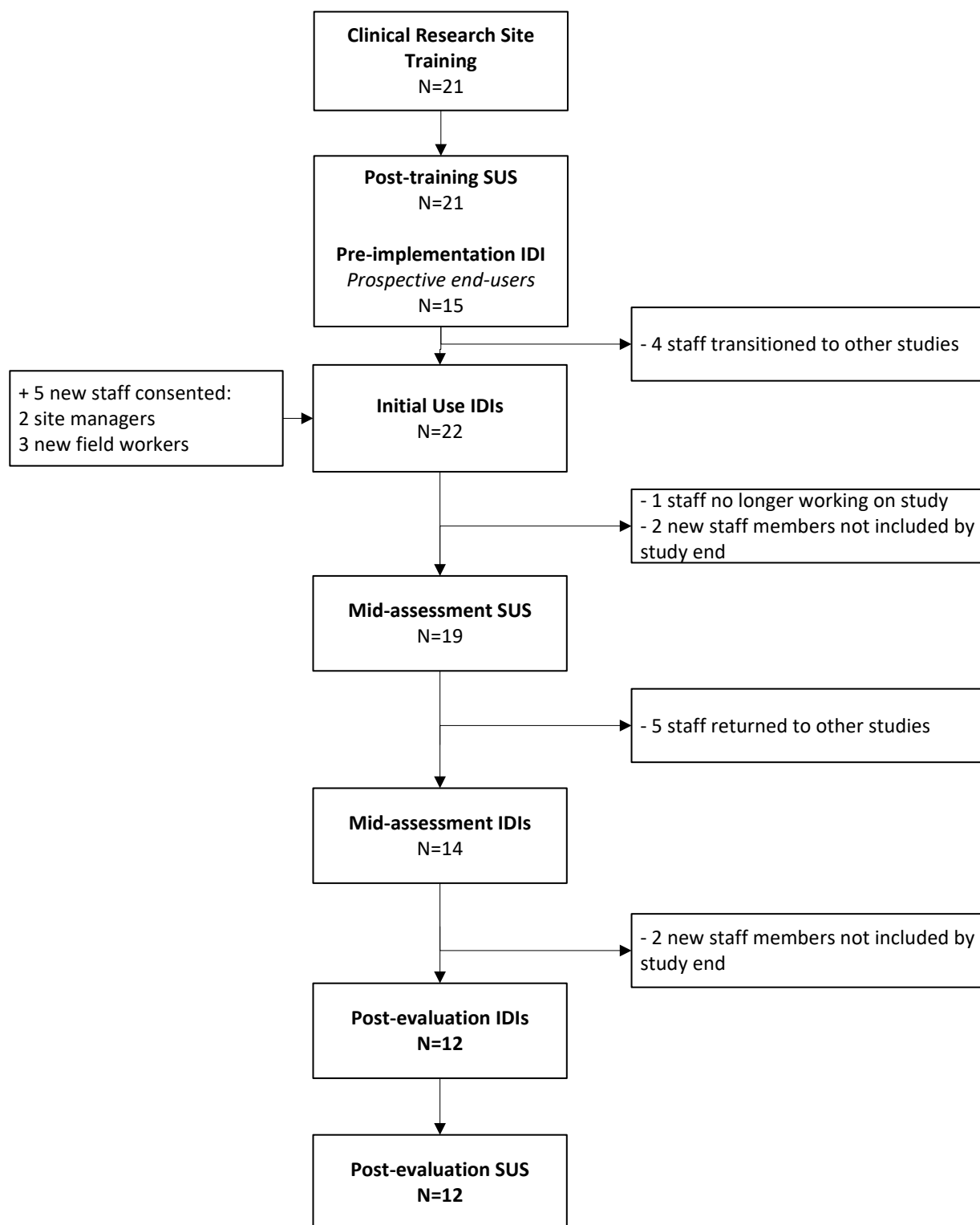

Supplement: S2 File — (PDF) [file pone.0286666.s002.pdf]
