# Supplementary material for: Usability of a novel lateral flow assay for the point-of-care detection of Neisseria gonorrhoeae: A qualitative time-series assessment among healthcare workers in South Africa
Source: PLoS One. 2023 Jun 2;18(6):e0286666. doi: 10.1371/journal.pone.0286666 (PMC10237465; doi:10.1371/journal.pone.0286666)
Supplement: Attachment — Submitted filename: Response to Reviewers_Revised data statement.docx [file pone.0286666.s003.docx]

| **Reviewer Comment** | **Author’s Revision** |
| --- | --- |
| **Journal Requirements** | |
| 1. Thank you for providing additional information regarding the regulations that apply to your data. We've made some final updates to your Data Availability Statement to conform to journal requirements. Can you please confirm whether the following proposed statement is accurate and suitable to appear alongside your manuscript?  “The dataset for the System Usability Scale (SUS) has been uploaded to the Open Science Framework data repository. The identifier for this dataset is DOI 10.17605/[OSF.IO/EFVHS](http://OSF.IO/EFVHS).  The qualitative data are not publicly available due to privacy or ethical restrictions as ensured by participant informed consent and South Africa’s protection of personal information act (POPIA).  The relevant contact for any data inquiries and access upon reasonable request is: Freedom Mukomana <[Freedomm@foundation.co.za](mailto:Freedomm@foundation.co.za)> Data Manager, Foundation for Professional Development" | Thank you for your revisions and consideration. We have taken note of the revised statement and confirm that the proposed statement is suitable for this manuscript. |
